# Author Correction: An optofluidic platform for interrogating chemosensory behavior and brainwide neural representation in larval zebrafish

**DOI:** 10.1038/s41467-023-38152-x

**Published:** 2023-05-05

**Authors:** Samuel K. H. Sy, Danny C. W. Chan, Roy C. H. Chan, Jing Lyu, Zhongqi Li, Kenneth K. Y. Wong, Chung Hang Jonathan Choi, Vincent C. T. Mok, Hei-Ming Lai, Owen Randlett, Yu Hu, Ho Ko

**Affiliations:** 1 Division of Neurology, Department of Medicine and Therapeutics, Faculty of Medicine, The Chinese University of Hong Kong, Shatin, New Territories, Hong Kong SAR, China; 2 Li Ka Shing Institute of Health Sciences, Faculty of Medicine, The Chinese University of Hong Kong, Shatin, New Territories, Hong Kong SAR, China; 3 Department of Biomedical Engineering, Faculty of Engineering, The Chinese University of Hong Kong, Shatin, New Territories, Hong Kong SAR, China; 4 Department of Electrical and Electronic Engineering, Faculty of Engineering, The University of Hong Kong, Pok Fu Lam, Hong Kong Island, Hong Kong SAR, China; 5 Advanced Biomedical Instrumentation Centre, Hong Kong Science Park, Pak Shek Kok, New Territories, Hong Kong SAR, China; 6 Department of Anaesthesia and Intensive Care, Faculty of Medicine, The Chinese University of Hong Kong, Shatin, New Territories, Hong Kong SAR, China; 7 Peter Hung Pain Research Institute, Faculty of Medicine, The Chinese University of Hong Kong, Shatin, New Territories, Hong Kong SAR, China; 8 Chow Yuk Ho Technology Centre for Innovative Medicine, The Chinese University of Hong Kong, Shatin, New Territories, Hong Kong SAR, China; 9 Margaret K. L. Cheung Research Centre for Management of Parkinsonism, Faculty of Medicine, The Chinese University of Hong Kong, Shatin, New Territories, Hong Kong SAR, China; 10 Gerald Choa Neuroscience Institute, The Chinese University of Hong Kong, Shatin, New Territories, Hong Kong SAR, China; 11 Department of Psychiatry, Faculty of Medicine, The Chinese University of Hong Kong, Shatin, New Territories, Hong Kong SAR, China; 12 Institut national de la santé et de la recherche médicale, Université Claude Bernard Lyon 1, Lyon, France; 13 Department of Mathematics and Division of Life Science, Faculty of Science, Hong Kong University of Science and Technology, Clear Water Bay, New Territories, Hong Kong SAR, China; 14 School of Biomedical Sciences, Faculty of Medicine, The Chinese University of Hong Kong, Shatin, New Territories, Hong Kong SAR, China

**Keywords:** Lab-on-a-chip, Light-sheet microscopy, Fluorescence imaging, Olfactory system, Neural circuits

Correction to: *Nature Communications* 10.1038/s41467-023-35836-2, published online 14 January 2023

The original version of this Article contained errors in Fig. 4d and Supplementary Fig. 4e, which showed plots of erroneous cumulative distance values, which arose from an erroneous handling of NaN values in the underlying code for distance calculations.

The correct version of Fig. 4d is:


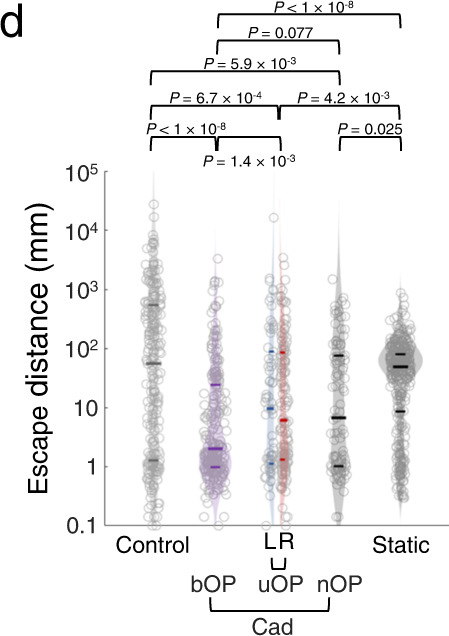


which replaces the previous incorrect version:


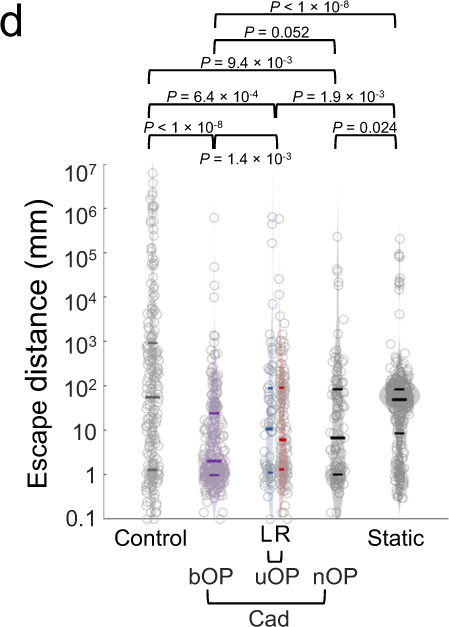


This has been corrected in both the PDF and HTML versions of the Article.

The correct version of Supplementary Fig. 4e is:


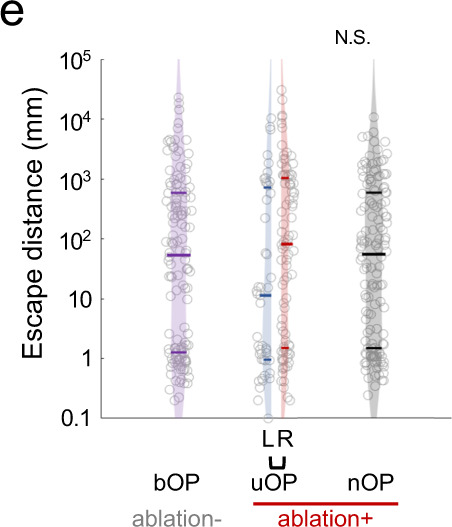


which replaces the previous incorrect version:


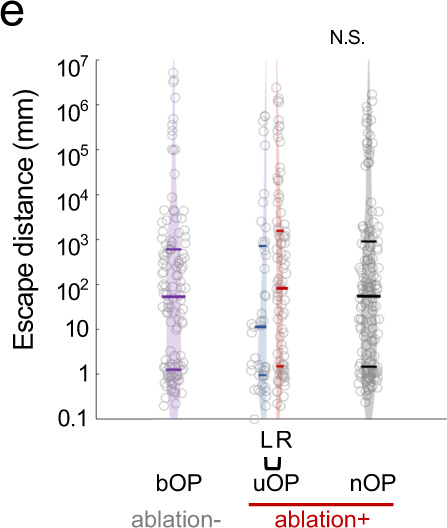


The HTML has been updated to include a corrected version of the Supplementary Information.

The Source Data file has also been corrected in the HTML version of the Article. The MATLAB code provided at the GitHub link in the Code Availability statement has been corrected.

In addition, in the results section “Integrated microfluidics-light sheet fluorescence microscopy system for imaging neuronal activities and behavior” the sentence “Simultaneously, the tail flipping behaviors were monitored using high-speed IR camera imaging at 200 fps (Fig. 3b)” incorrectly referred to Fig. 3b, instead of Fig. 3c, and in the results section “Revealing brainwide representation of cadaverine sensing using the integrated optofluidic system” the sentence “Interestingly, apart from observing a supralinear gain of information for the majority of neurons (Fig. 5b, g–i), there is a trend for F_*Is*_ to increase along the rostral-caudal axis (Fig. 5h, i) as I_*S*_ values decrease across the forebrain regions (Fig. 5a)” incorrectly referred to Fig. 5b, g–i, instead of Fig. 5c, g–i. These errors have been corrected in both the PDF and the HTML versions of the Article.

## Supplementary information

Updated Supplementary Information

Updated Source Data

