# Supplementary material for: An optofluidic platform for interrogating chemosensory behavior and brainwide neural representation in larval zebrafish
Source: Nat Commun. 2023 Jan 14;14:227. doi: 10.1038/s41467-023-35836-2 (PMC9840631; doi:10.1038/s41467-023-35836-2)
Supplement: Supplementary file 1 — Supplementary Information [file 41467_2023_35836_MOESM1_ESM.pdf]

Supplementary Information

An optofluidic platform for interrogating chemosensory behavior and brainwide neural representation in larval zebrafish

Sy et al.

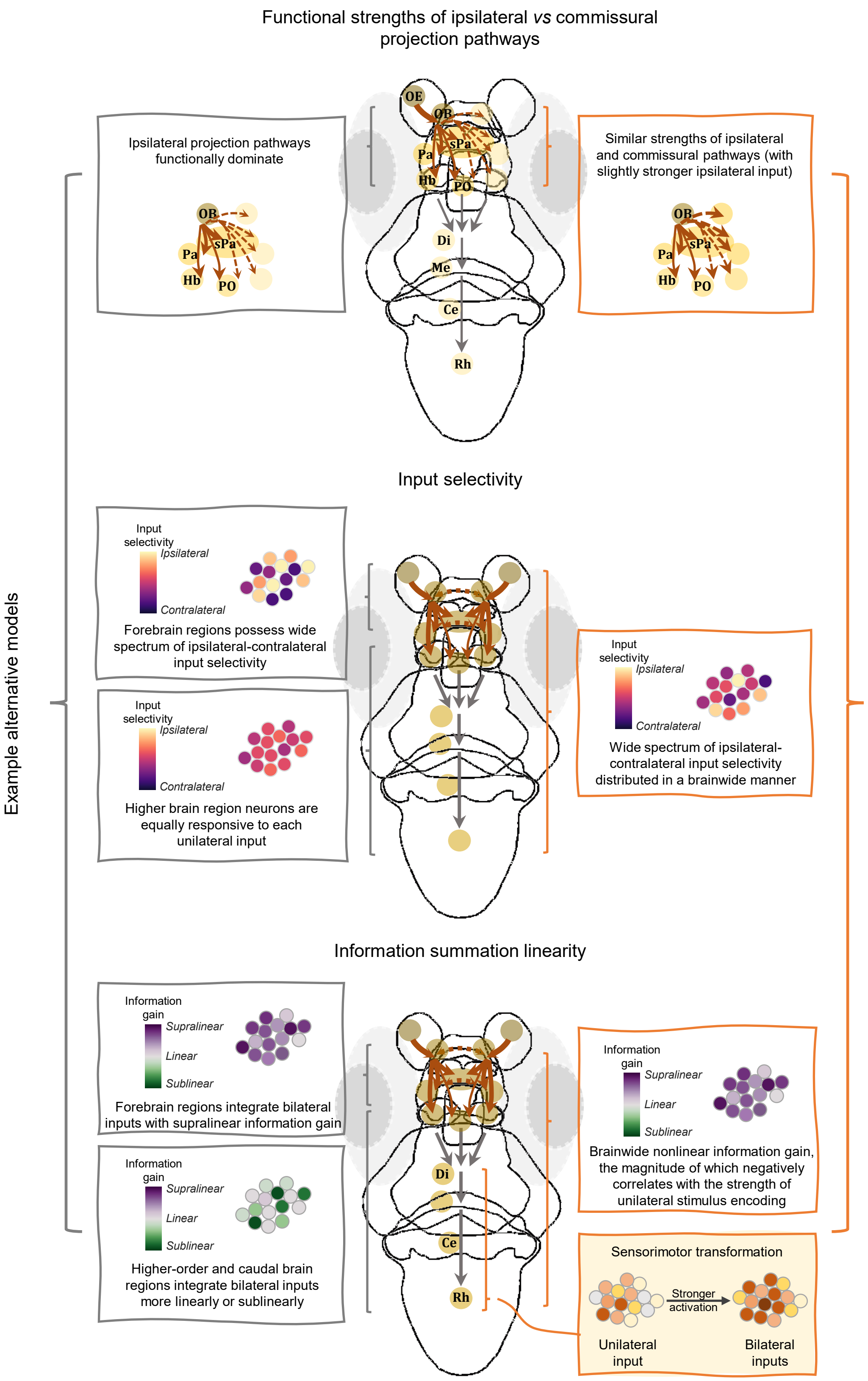

Supplementary Fig. 1. Circuit models for bilateral olfactory input integration in the larval zebrafish. Examples of alternative circuit processing models (left panels) compared to that constrained by our functional imaging data (right panels), to account for binasal input-mediated sensory processing and sensorimotor transformation in cadaverine avoidance.

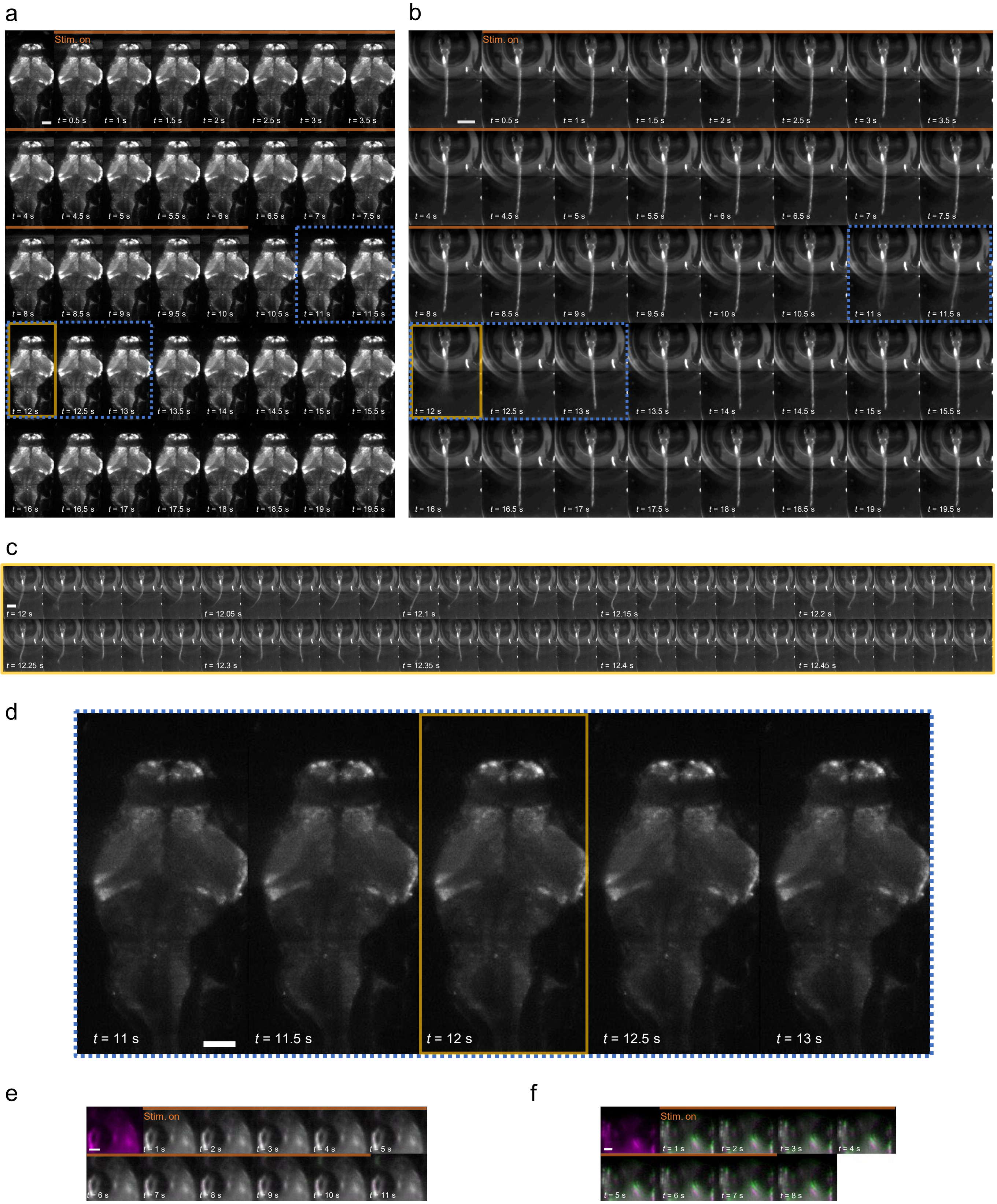

Supplementary Fig. 2. Stable cellular resolution imaging during tail flipping and stimulus delivery. (a) Time-series neuronal GCaMP6f fluorescence images of a plane from an example larva. Imaging frames during stimulus delivery (Stim. on) are indicated. The chemical streams for bilateral olfactory stimulation can be seen by sodium fluorescein fluorescence in front of the larva for these frames. Scale bar: 100 µm. **(b)** Simultaneously acquired time-series tail-flipping images (averaged every 0.5 s) from the same larva in **(a)**. **(c)** Time-series images (at a higher frame rate, averaged every 10 ms) of rigorous tail flipping for the same period as the outlined (solid yellow line) frames in **(a)** and **(b)**. Scale bars in **(b)** and **(c)**: 1 mm. **(d)** Magnified neuronal images corresponding to the period outlined (dashed blue line) in **(a)** and **(b)**. **(e)** Time-series (averaged every 1 s) mean intensity projection images of a part of the head of the larva in **(a)**, with each image frame from the second one onwards overlaid over the first as reference (current frame in green and reference image in magenta). **(f)** Similar to **(e)** but from another experiment using a µfluidic device without side channel to dissipate pressure changes. Scale bars in **(d)**, **(e)** and **(f)**: 100 µm.

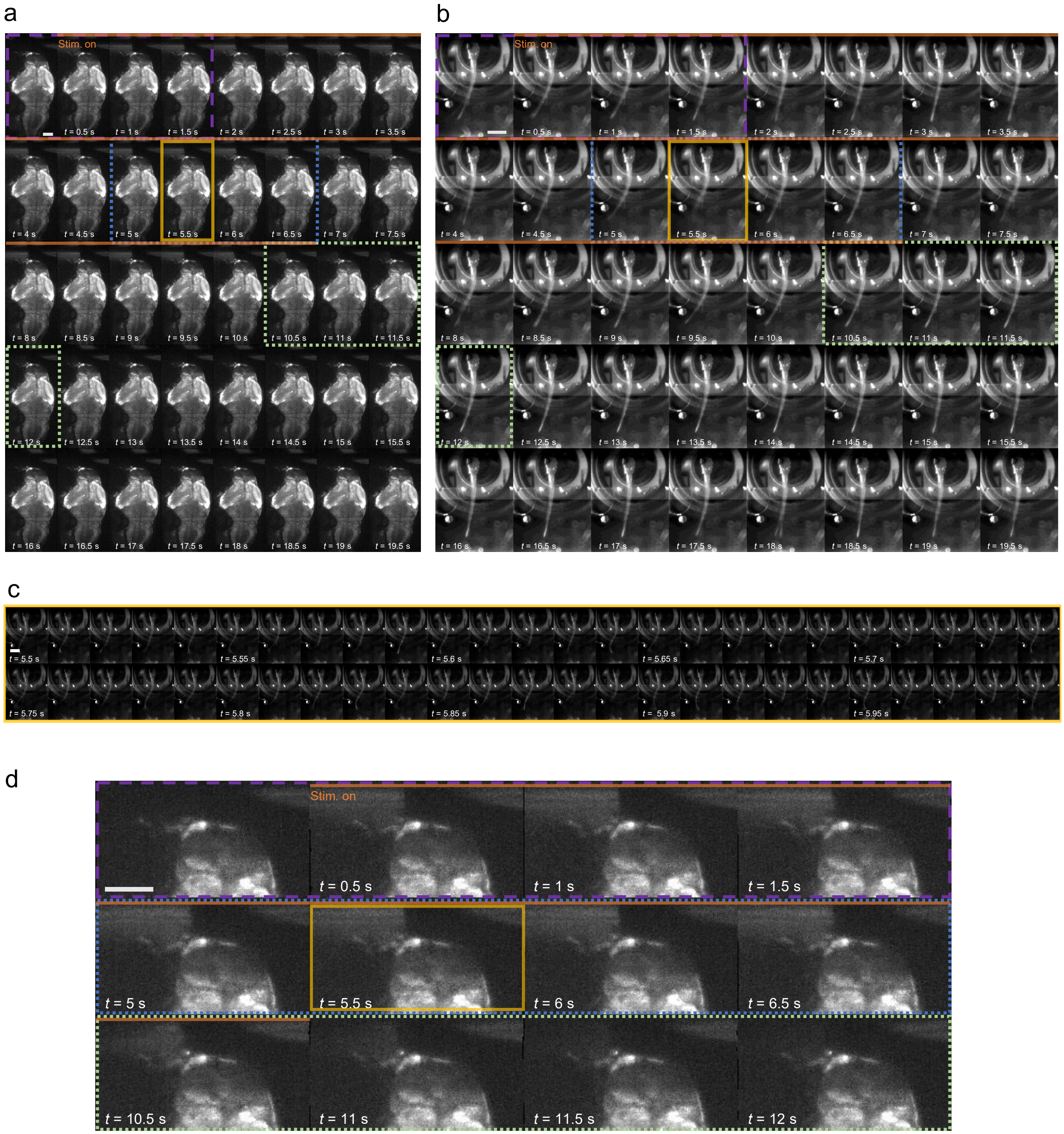

Supplementary Fig. 3. Further examples of stable cellular resolution imaging during tail flipping and stimulus delivery. (a) Time-series neuronal GCaMP6f fluorescence images of a plane from an example larva. Imaging frames during stimulus delivery (Stim. on) are indicated. The chemical streams for left olfactory stimulation can be seen by sodium fluorescein fluorescence in front of the larva for these frames. Scale bar: 100 µm. **(b)** Simultaneously acquired time-series tail-flipping images (averaged every 0.5 s) from the same larva in **(a)**. **(c)** Time-series images (at a higher frame rate, averaged every 10 ms) of rigorous tail flipping for the same period as the outlined (solid yellow line) frames in **(a)** and **(b)**. Scale bars in **(b)** and **(c)**: 1 mm. **(d)** Magnified neuronal images of a part of larval forebrain corresponding to the periods outlined (dashed magenta, blue, and green) in **(a)** and **(b)**. Scale bar: 100 µm.

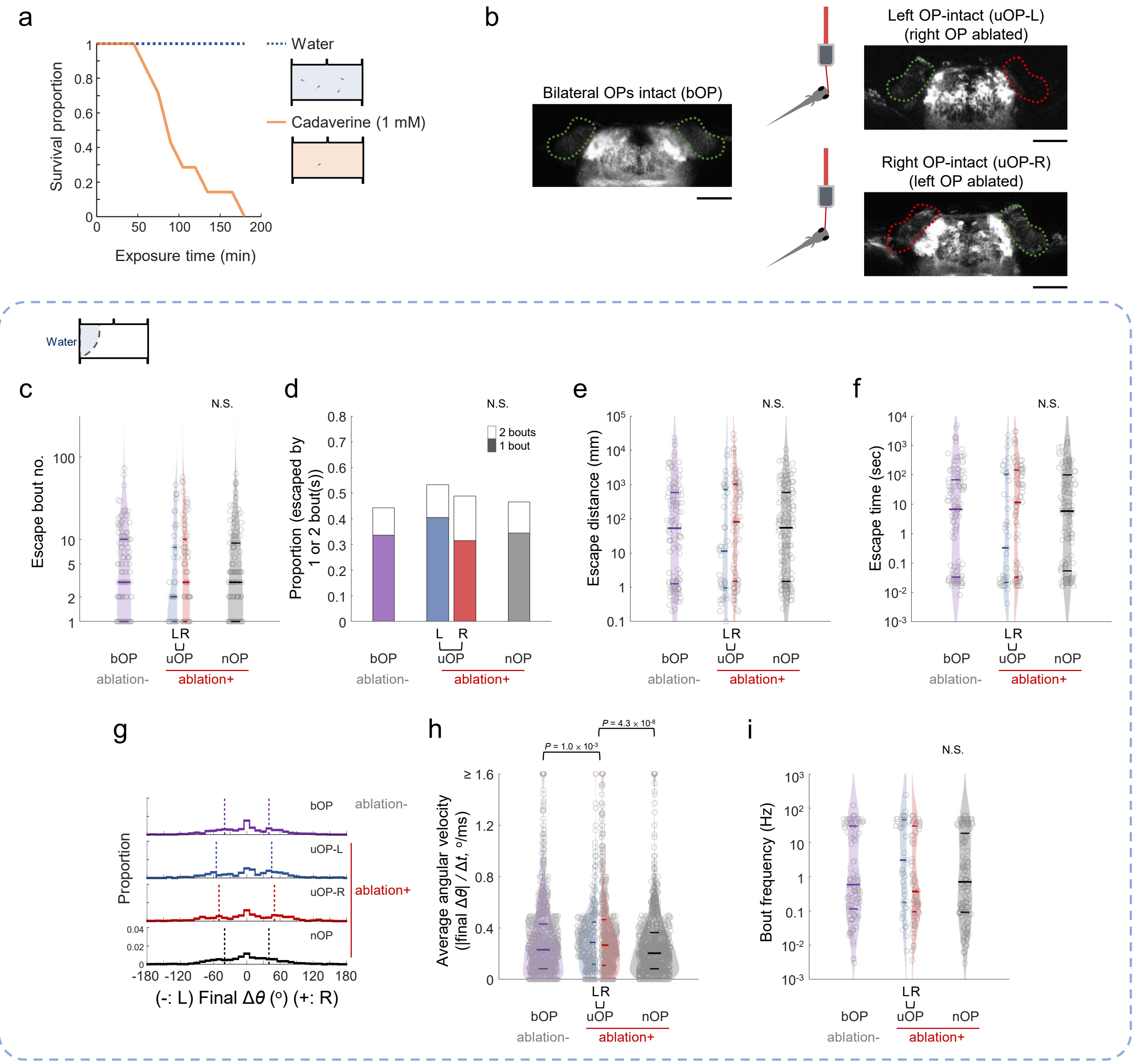

Supplementary Fig. 4. Survival assay, schematics of two-photon ablation, and control analysis of swim behavior for the ablated groups in the mirror zone of avoidance assays. (a) Proportion of larvae surviving in an arena same as the microfluidic swimming arena filled by just cadaverine (1 mM in water, n = 7, orange solid line) or just water (control, n = 6, blue dotted line) with time. **(b)** Left panel: Two-photon image of a zebrafish larva with the olfactory placodes (OPs) outlined by green dotted lines (designated as bilateral OP-intact, or bOP larvae). Right panel: Images of example unilateral OP-intact (uOP) larvae with two-photon ablation of the right OP (upper image: left OP intact or uOP-L) or left OP (lower image: right OP intact or uOP-R). Intact OPs are outlined in green, and ablated OPs are outlined in red dotted lines. Scale bars: 50 µm. **(c) – (f)** Performance metrics of "escape" journeys in the mirror zone (i.e., from entering to leaving the mirror zone), including **(c)** bout number, **(d)** proportion of entry-to-exit events with only 1 or 2 bouts (N.S.: Non-significant with Chi-squared test comparing 2-bout event proportions), **(e)** distance travelled, and **(f)** time taken to leave the mirror zone in avoidance behavioral assays (bOP larvae without ablation (ablation-), left OP-intact (L) or right OP-intact (R) uOP larvae, and null OP-intact (nOP larvae) that had undergone ablation (ablation+) in arenas with cadaverine stream in the stimulus zone). **(g)** Histograms of turn angle distributions of bouts (i.e., final $\Delta\theta$), **(h)** Average angular velocity of bouts (i.e., |final $\Delta\theta$|/Δt), and **(i)** Swim bout frequency in the mirror zone. In **(c)**, **(e)**, **(f)** and **(i)**, the parameters are plotted in log scales. In **(c)**, **(e)**, **(f)**, **(h)** and **(i)**: Horizontal lines indicate the medians, 75 and 25 percentiles for each group. Shadows of the violin plots scale according to the probability density function. P-values: Kruskal–Wallis test with Tukey's post-hoc test. N.S.: Non-significant. For post-hoc tests in **(h)**, only comparisons with significant P-values marked. In **(c)**–**(i)**, numbers of mirror zone border-crossing events: bOP: 137; uOP-L: 49; uOP-R: 92; nOP: 145. Source data are provided as a Source Data file.

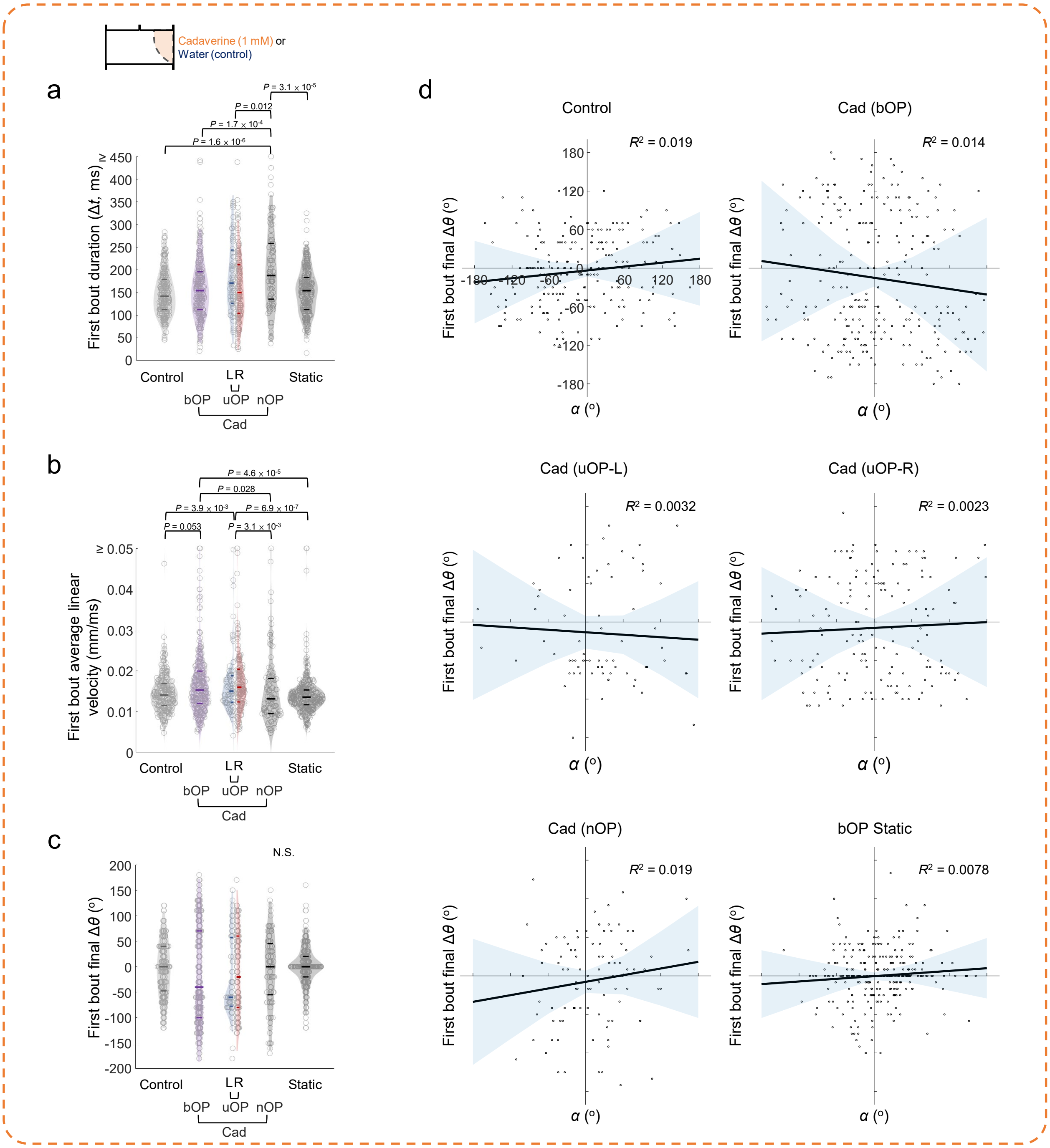

Supplementary Fig. 5. Additional kinematic parameters upon cadaverine encounter in the chemical zones of avoidance assays. (a) First bout duration. **(b)** First bout average linear velocity. **(c)** Final $\Delta\theta$ for the first bouts. **(d)** First turn final $\Delta\theta$ plotted against incidence angle (α) on zone entry (also see **Fig. 4f**). In **(a)**, **(b)** and **(c)**: Horizontal lines indicate the medians, 75 and 25 percentiles for each group. Shadows of the violin plots scale according to the probability density function. *P*-values: Kruskal–Wallis test with Tukey's post-hoc test. N.S.: Non-significant. Numbers of assays, larvae, and rightmost zone border-crossing events: bOP (control): 6, 24, 211; bOP (avoidance): 8, 32, 251; uOP-L: 4, 15, 71; uOP-R: 4, 15, 155; nOP: 7, 23, 96; bOP Static: 3, 11, 283. Source data are provided as a Source Data file.

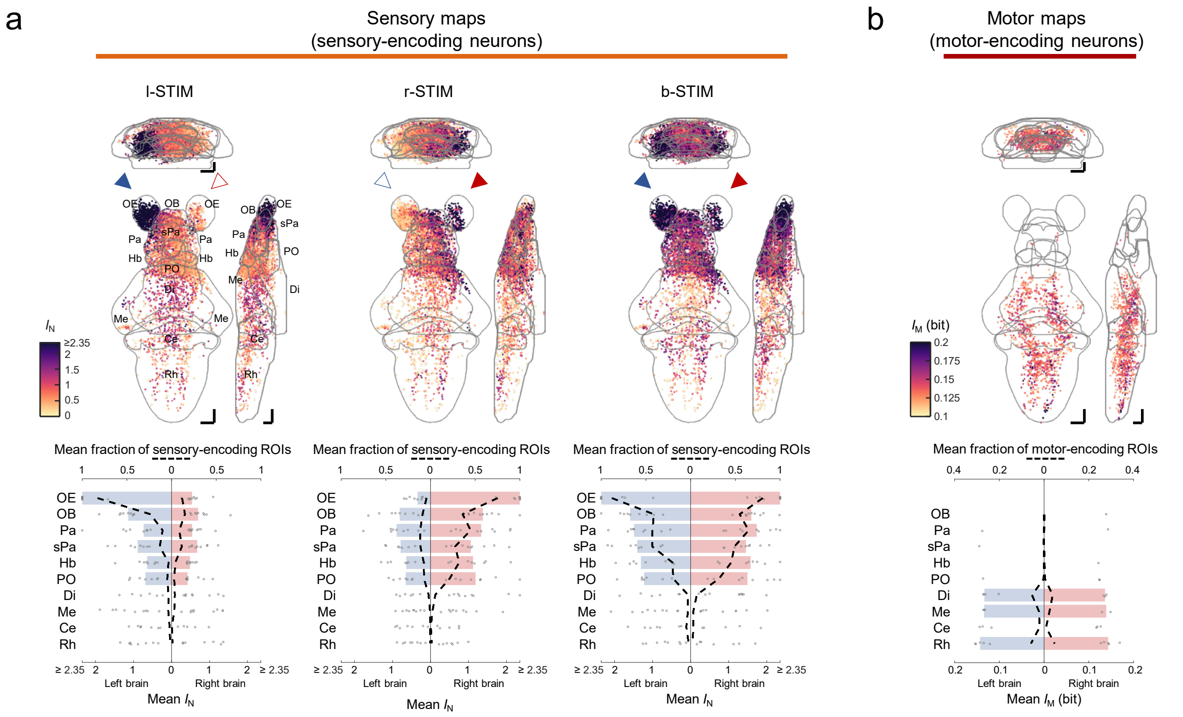

Supplementary Fig. 6. Brainwide activity maps evoked by cadaverine stimulation. (a) Upper panels: Mean intensity projections of the normalized mutual information between the calcium signals of ROIs and cadaverine stimulus profile of l-STIM (left panel), r-STIM (middle panel) or b-STIM (right panel) pooled across larvae (*n* = 9) (I_N of sensory-encoding ROIs). Solid triangles mark the corresponding OP(s) stimulated. Lower panels: The distributions of mean I_N of sensory-encoding ROIs in the different brain regions during l-STIM (left panel), r-STIM (middle panel) or b-STIM (right panel) among the larvae (*n* = 9). Regions with top six mean fractions of sensory-encoding ROIs with b-STIM are OE, OB, Pa, sPa, Hb and PO. Bars represent the medians of the mean I_N of sensory-encoding ROIs in these regions. Dashed lines indicate mean fractions of sensory-encoding ROIs in the different regions among the larvae. **(b)** Upper panel: Mean intensity projections of mutual information between the calcium signals of ROIs and tail flipping frequency (I_M of motor-encoding ROIs) pooled across larvae (*n* = 6). Regions with top three fractions of motor-encoding ROIs are Di, Me and Rh. Lower panel: The distributions of I_M of motor-encoding ROIs in the different brain regions pooled across larvae (*n* = 6), with bars representing the medians of mean I_M of motor-encoding ROIs, and dashed lines indicating mean fractions of motor-encoding ROIs, among the larvae. Abbreviations of brain regions: same as in **Fig. 3e**. Scale bars in **(a)** and **(b)**: 50 μm in Z-Brain atlas space. Source data are provided as a Source Data file.

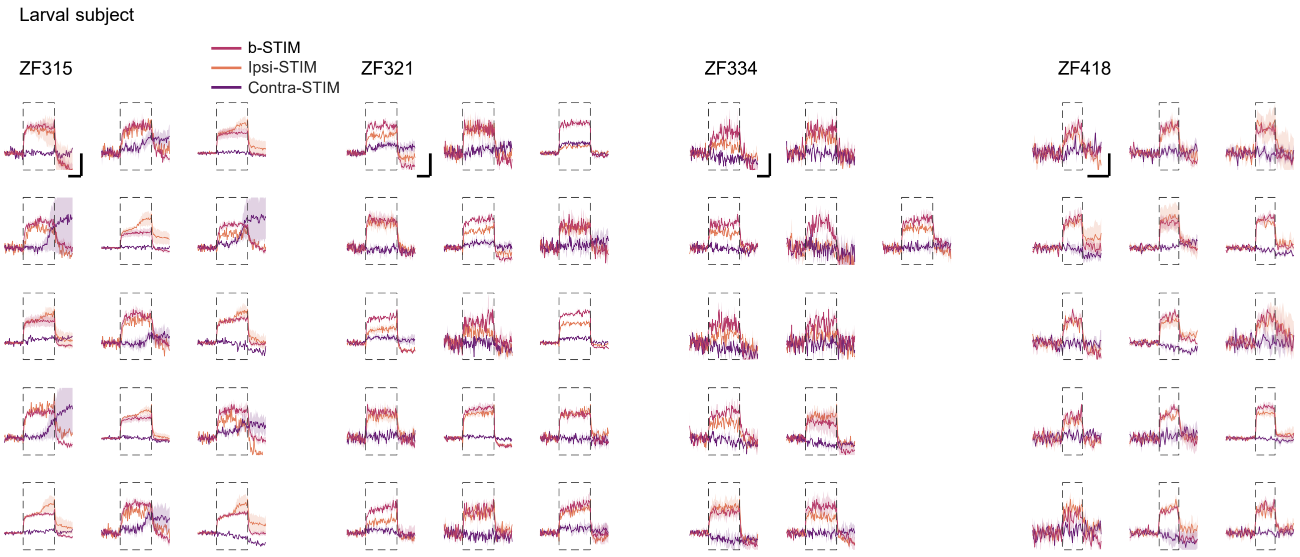

Supplementary Fig. 7. Further examples of cadaverine-responsive olfactory sensory neurons (OSNs). More example trial-averaged responses to ipsilateral (ipsi-STIM, orange), contralateral (contra-STIM, violet) or bilateral (b-STIM, cherry) olfactory stimulation of individual regions-of-interest (ROIs) corresponding to cadaverine-responsive olfactory sensory neurons (OSNs) in the olfactory epithelia (OE) from four imaged larval zebrafish. For larval subjects ZF315, ZF321 and ZF418, 15 OSNs were randomly sampled and shown, whereas ZF334 had only 11 cadaverine-responsive OSNs detected. Shadow shows SEM across trials for each trace. Dashed rectangle indicates stimulus window. Scale bars: 10 seconds (horizontal) and 0.5 normalized dF/F (vertical).

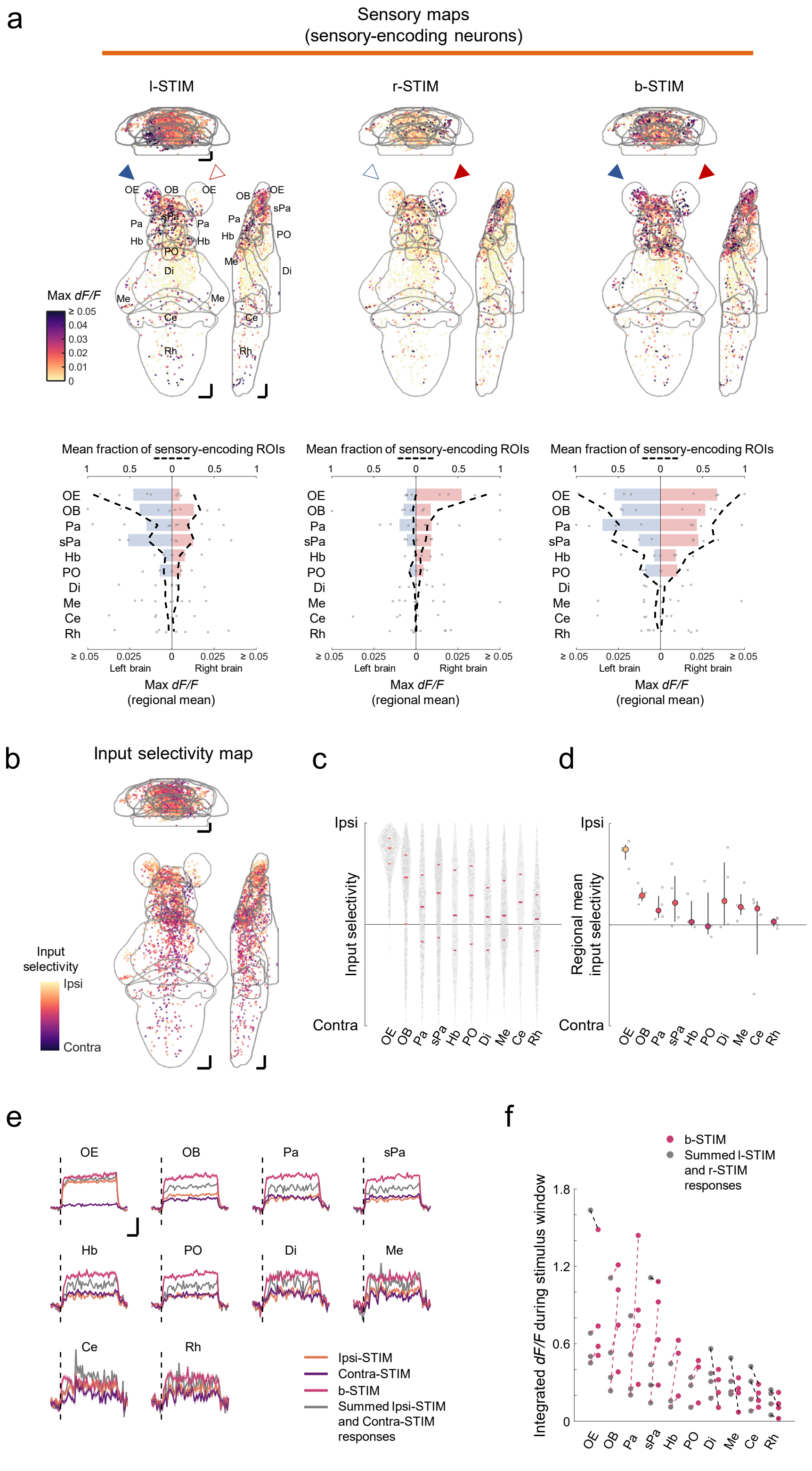

Supplementary Fig. 8. Brainwide activity and binasal input integration rules quantified with GCaMP6f fluorescence changes from baseline (*dF*/*F*). **(a)** Upper panels: Mean intensity projections (to coronal, transverse and sagittal planes) of maximum *dF*/*F* over stimulus time windows, for sensory-encoding regions-of-interest (ROIs) during l-STIM (left panel), r-STIM (middle panel) or b-STIM (right panel) trials, pooled from 4 larvae with unbiased responses to l-STIM or r-STIM. Solid triangles mark the corresponding OP(s) stimulated. Abbreviations of brain regions: same as in **Fig. 3e.** Lower panels: Brainwide distributions of regional mean of maximum *dF*/*F* related to the projections. Dashed lines indicate mean fractions among the larvae. Bars representing the medians in regions with top six mean fraction with b-STIM. Scale bars: 50 µm in Z-Brain atlas space. **(b)** Mean intensity projection maps (to coronal, transverse and sagittal planes) of ipsilateral(Ipsi)-contralateral(Contra) input selectivity (quantified from integrated *dF*/*F* over stimulus windows for the sensory-encoding ROIs). Scale bars: 50 µm in Z-Brain atlas space. **(c)** Brainwide distributions of Ipsi-Contra input selectivity. Lines represent medians, 75 and 25 percentiles for each brain region. Shadows of the violin plots scale according to the probability density function. **(d)** Regional means of Ipsi-Contra input selectivity, with each small dot representing the value from one larva. Large dots, upper and lower limits of lines: medians, 75 and 25 percentiles, respectively. **(e)** Regionally averaged stimulus-locked neuronal responses with ipsilateral (Ipsi-STIM, orange), contralateral (Contra-STIM, violet), bilateral cadaverine stimulation (b-STIM, cherry), and linearly summed regional average responses to Ipsi-STIM and Contra-STIM (grey), from one example larva. Vertical dashed lines indicate stimulus onset. Scale bars: 5 seconds (horizontal), and 0.5 normalized *dF*/*F* (vertical). Shadows: SEM. **(f)** Integrated regionally averaged neuronal responses (i.e., summed *dF*/*F* over stimulus window) to bilateral cadaverine stimulation (b-STIM, cherry), and linearly summed regional average responses to l-STIM and r-STIM (grey). Each pair of connected dots represents data from one larval zebrafish. Sign of difference (b-STIM response – (l-STIM response + r-STIM response)) is represented by the color of connecting dashed line (cherry and grey for positive and negative signs, respectively). In **(b)**, **(c)**, **(d)** and **(f)**, data are pooled across the same larvae group as in **(a)** and **Fig. 5d–i**. Source data are provided as a Source Data file.

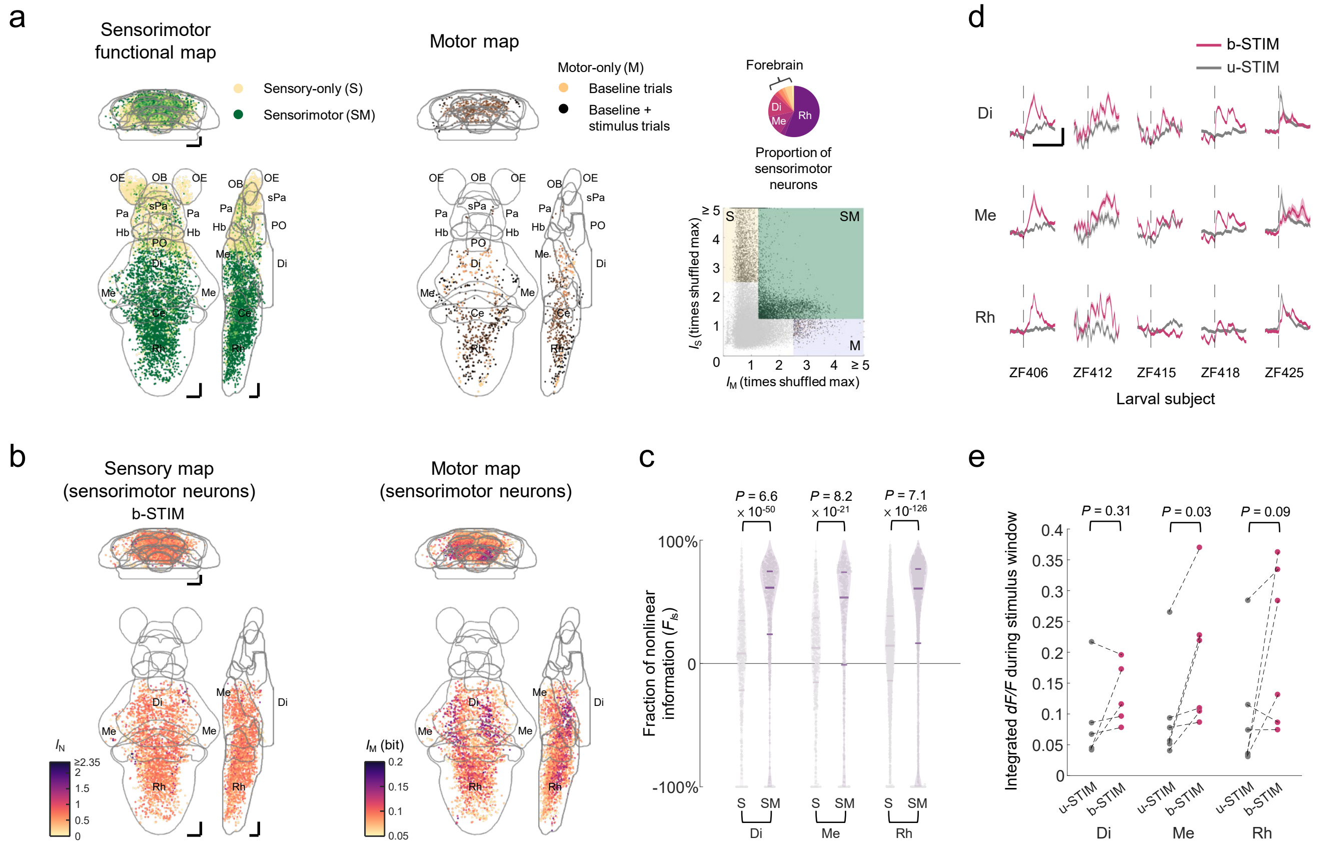

Supplementary Fig. 9. Binasal input-dependent activation of sensorimotor and motor units in cadaverine sensing. (a) Left panel: Whole-brain functional maps (projections to coronal, transverse and sagittal planes) of sensory-only (S) and sensorimotor (SM) ROIs pooled across the behaviorally responsive and stably imaged larvae (n = 6). Middle panel: Whole-brain maps of baseline trial-motor-encoding (baseline trials) and all trial-motor-encoding (baseline + stimulus trials) ROIs. Right upper panel: Pie chart showing the distribution of sensorimotor ROIs in the different brain regions. Right lower panel: Scatter plot showing the distributions of the mutual information between the calcium signals of each ROI and cadaverine stimulus profile (maximum I_S of l-STIM, r-STIM and b-STIM conditions), and tail flipping frequency (I_M), both calculated as the number of times the maximum of the corresponding shuffled values (see **Methods**). Abbreviations of brain regions: same as in **Fig. 3e**. Scale bars: 50 µm in Z-Brain atlas space. **(b)** Mean intensity projection maps of the mutual information between the calcium signals of sensorimotor ROIs and cadaverine stimulus profile of b-STIM (left panel, normalized mutual information I_N) and tail flipping frequency (right panel, mutual information I_M), in Di, Me and Rh pooled across the same group of larvae. Scale bars: 50 µm in Z-Brain atlas space. **(c)** Distributions of the fraction of nonlinear information (F_{I_S}) of sensory-only (S) and sensorimotor (SM) ROIs in Di, Me and Rh, drawn from same dataset as in **(a)**. Horizontal lines: medians, 75 and 25 percentiles. Shadows of the violin plots scale according to the probability density function. P-values: Two-sided Wilcoxon rank-sum test. **(d)** Regionally averaged stimulus-locked sensorimotor neuronal responses by bilateral (b-STIM, cherry) and unilateral (u-STIM, grey) cadaverine stimulation from 5 larvae with sensorimotor ROIs identified in all three regions. Vertical dashed line indicates stimulus onset. Scale bars: 10 seconds (horizontal) and 0.5 normalized dF/F (vertical) from each larval subject. Shadows: SEM. **(e)** Integrated regionally averaged sensorimotor neuronal responses (i.e., summed dF/F over stimulus window) to b-STIM (cherry) and u-STIM (grey). Each pair of connected dots represents data from one larval zebrafish. P-values: Two-sided Wilcoxon signed-rank test. Source data are provided as a Source Data file.
