# Supplementary material for: An optofluidic platform for interrogating chemosensory behavior and brainwide neural representation in larval zebrafish
Source: Nat Commun. 2023 Jan 14;14:227. doi: 10.1038/s41467-023-35836-2 (PMC9840631; doi:10.1038/s41467-023-35836-2)
Supplement: Supplementary file 3 — Reporting Summary [file 41467_2023_35836_MOESM3_ESM.pdf]

nature portfolio

Corresponding author(s): Ho Ko
Last updated by author(s): Dec 5, 2022

Reporting Summary

Nature Portfolio wishes to improve the reproducibility of the work that we publish. This form provides structure for consistency and transparency in reporting. For further information on Nature Portfolio policies, see our Editorial Policies and the Editorial Policy Checklist.

Statistics

For all statistical analyses, confirm that the following items are present in the figure legend, table legend, main text, or Methods section.

n/a	Confirmed	
☐	☒	The exact sample size (*n*) for each experimental group/condition, given as a discrete number and unit of measurement
☐	☒	A statement on whether measurements were taken from distinct samples or whether the same sample was measured repeatedly
☐	☒	The statistical test(s) used AND whether they are one- or two-sided<br>*Only common tests should be described solely by name; describe more complex techniques in the Methods section.*
☐	☒	A description of all covariates tested
☐	☒	A description of any assumptions or corrections, such as tests of normality and adjustment for multiple comparisons
☐	☒	A full description of the statistical parameters including central tendency (e.g. means) or other basic estimates (e.g. regression coefficient) AND variation (e.g. standard deviation) or associated estimates of uncertainty (e.g. confidence intervals)
☐	☒	For null hypothesis testing, the test statistic (e.g. *F*, *t*, *r*) with confidence intervals, effect sizes, degrees of freedom and *P* value noted<br>*Give P values as exact values whenever suitable.*
☒	☐	For Bayesian analysis, information on the choice of priors and Markov chain Monte Carlo settings
☒	☐	For hierarchical and complex designs, identification of the appropriate level for tests and full reporting of outcomes
☐	☒	Estimates of effect sizes (e.g. Cohen's *d*, Pearson's *r*), indicating how they were calculated

Our web collection on statistics for biologists contains articles on many of the points above.

Software and code

Policy information about availability of computer code

Data collection	The behavioral data were acquired with Streampix 7 (Norpix, Canada).<br>The neuronal imaging data were acquired with custom codes written with Andor SDK3 for MATLAB, in MATLAB (R2017b).
Data analysis	The behavioral data were processed with custom code written in MATLAB (R2018b).<br>Computational fluid dynamics simulation were performed using CFDTool toolbox (Precision Simulation) (Version 1.4.0; https://www.mathworks.com/matlabcentral/fileexchange/72640-cfdtool-matlab-cfd-simulation-gui-toolbox).<br>Anatomical stacks were registered to the Z-Brain Atlas using custom scripts written in MATLAB (R2018b).<br>Stripe artifacts in the anatomical stacks and functional imaging frames were removed using the Variational Stationary Noise Remover (VSNR) algorithm (only one version available; obtained from https://www.math.univ-toulouse.fr/~weiss/Codes/VSNR/VSNR.zip).<br>Functional imaging frames were motion-corrected using the NoRMCorrE algorithm (only one version available; obtained from https://github.com/flatironinstitute/NoRMCorre).<br>Regions of interest (ROIs) corresponding to individual neurons were then extracted using the CaImAn package (Version 1.0.0.0; https://github.com/flatironinstitute/CaImAn-MATLAB) and analyzed with custom codes written in MATLAB (R2018b).<br><br>The custom MATLAB codes for data analysis are available at [https://github.com/khsamuelsy/FishChip].

For manuscripts utilizing custom algorithms or software that are central to the research but not yet described in published literature, software must be made available to editors and reviewers. We strongly encourage code deposition in a community repository (e.g. GitHub). See the Nature Portfolio guidelines for submitting code & software for further information.

Data

Policy information about availability of data

All manuscripts must include a data availability statement. This statement should provide the following information, where applicable:

- Accession codes, unique identifiers, or web links for publicly available datasets
- A description of any restrictions on data availability
- For clinical datasets or third party data, please ensure that the statement adheres to our policy

The AutoCAD files of the devices developed in this study are available at [https://doi.org/10.5281/zenodo.7355156]. Preprocessed data necessary to replicate these results are available at [https://doi.org/10.5281/zenodo.7306139]. Source data are provided with this paper.

Human research participants

Policy information about studies involving human research participants and Sex and Gender in Research.

Reporting on sex and gender	Not applicable.
Population characteristics	Not applicable.
Recruitment	Not applicable.
Ethics oversight	Not applicable.

Note that full information on the approval of the study protocol must also be provided in the manuscript.

Field-specific reporting

Please select the one below that is the best fit for your research. If you are not sure, read the appropriate sections before making your selection.

☒ Life sciences ☐ Behavioural & social sciences ☐ Ecological, evolutionary & environmental sciences

For a reference copy of the document with all sections, see nature.com/documents/nr-reporting-summary-flat.pdf

Life sciences study design

All studies must disclose on these points even when the disclosure is negative.

Sample size	This study did not involve prior sample calculation. Instead, we decided on the sample sizes based on the usual requirements for the specific types of experiments, taking reference to previous similar studies (e.g., Herrera et al., Curr. Biol., 2021; Koide et al., Cell Rep., 2018; Krishnan et al., Curr. Biol., 2014),
Data exclusions	For the behavioral assays, no data was excluded.<br>For the imaging experiments with unilateral olfactory stimulation intended, trials with > 1/20 spillover were excluded; trials with seizure-like brain activity and/or unsatisfactory image registration were also excluded from further analysis.
Replication	Repeating independent batches of experiments was the major means to ensure reproducibility (i.e., instead of relying on the data from a single experiment or animal). The reproducibility of experiments are evident from the respective plots showing summary statistics and error bars.<br>For the chemosensory avoidance navigation assay, the numbers of independent assays, larvae and border-crossing event sample sizes for the six swimming arena experimental groups are as follows: bilateral OP-intact larvae (control group without cadaverine zone, i.e., all water zones): 6, 24, 211; bilateral OP-intact larvae (assay group with cadaverine zone): 8, 32, 251; left OP-intact larvae (assay group with cadaverine zone): 4, 15, 71; right OP-intact larvae (assay group with cadaverine zone): 4, 15, 155; null OP-intact larvae (assay group with cadaverine zone): 7, 23, 96; bilateral OP-intact static (all water control without flow): 3, 11, 283.<br>For the brain imaging assay, eighteen 5–6 d.p.f. right eye-ablated and four right-tilted zebrafish larvae underwent simultaneous behavioral and whole-brain calcium imaging experiments, among which nine exhibited spontaneous tail flipping in the µfluidic device (all right eye-ablated), and we obtained stable imaging from nine larvae (6 right eye-ablated and 3 right-tiled). These data were used for analysis of neural activity evoked by unilateral and bilateral cadaverine stimulation.
Randomization	Since our experiments did not include comparisons across heterogeneous samples subject to different treatment effects, randomization was not relevant to our study design.
Blinding	Since our experiments did not include obtaining readouts that may be affected by the experimenter's knowledge of sample group, and all analyses were based on identical software pipelines, blinding was not necessary for our study.

Reporting for specific materials, systems and methods

We require information from authors about some types of materials, experimental systems and methods used in many studies. Here, indicate whether each material, system or method listed is relevant to your study. If you are not sure if a list item applies to your research, read the appropriate section before selecting a response.

Materials & experimental systems

n/a	Involved in the study
☒	☐ Antibodies
☒	☐ Eukaryotic cell lines
☒	☐ Palaeontology and archaeology
☐	☒ Animals and other organisms
☒	☐ Clinical data
☒	☐ Dual use research of concern

Methods

n/a	Involved in the study
☒	☐ ChIP-seq
☒	☐ Flow cytometry
☒	☐ MRI-based neuroimaging

Animals and other research organisms

Policy information about studies involving animals; ARRIVE guidelines recommended for reporting animal research, and Sex and Gender in Research

Laboratory animals	For all experiments reported in this study, we used 5–7 days post fertilization (d.p.f.) larvae carrying the nacre mutation bred from individual pairs of heterozygous Tg(elavl3:GCaMP6f) adult zebrafish.
Wild animals	This study did not involve wild animals.
Reporting on sex	Sex was not determined for the larvae at this developmental stage (5–7 days post fertilization).
Field-collected samples	This study did not involve field-collected samples.
Ethics oversight	All experimental procedures were approved in advance by the Animal Research Ethical Committee of the Chinese University of Hong Kong and were carried out in accordance with the Guide for the Care and Use of Laboratory Animals.

Note that full information on the approval of the study protocol must also be provided in the manuscript.
